# An Investigation into Smartphone Addiction with Personality and Sleep Quality among University Students

**DOI:** 10.3390/ijerph18147588

**Published:** 2021-07-16

**Authors:** Hsien-Yuan Lane, Chin-Jui Chang, Chieh-Liang Huang, Yun-Hsuan Chang

**Affiliations:** 1 Graduate Institute of Biomedical Sciences, China Medical University, Taichung 404332, Taiwan; hylane@mail.cmuh.org.tw; 2 Department of Psychiatry & Brain Disease Research Center, China Medical University Hospital, Taichung 404332, Taiwan; 3 Department of Psychology, College of Medical and Health Science, Asia University, Taichung 41354, Taiwan; 4 Department of Public Policy and Administration, National Chi Nan University, Nantou 54561, Taiwan; changgary211@ncnu.edu.tw; 5 Department of Psychiatry, Tsaotun Psychiatric Center, Ministry of Health and Welfare, Nantou 54249, Taiwan; chlhuang@ttpc.mohw.gov.tw; 6 Department of Optometry, Asia University, Taichung 41354, Taiwan; 7 Center for Internet Addiction Prevention and Intervention, Asia University, Taichung 41354, Taiwan; 8 Clinical Psychological Center, Asia University Hospital, Taichung 41354, Taiwan; 9 Department of Medical Research, China Medical University Hospital, Taichung 404332, Taiwan

**Keywords:** harm avoidance, novelty seeking, personality, sleep quality, smartphone addiction, vulnerability

## Abstract

Over the past two decades, smartphones have become common, and the accompanying devices have also become much more popular and easily accessible worldwide. With the development of smartphones, accompanied by internet facilities, excessive smartphone use or smartphone addiction may cause sleep disturbance and daily dysfunction. This study proposed examining the association between personality traits and smartphone addiction and its effects on sleep disturbance. Four hundred and twenty-two university participants (80 male and 342 female participants) with a mean age of 20.22 years old were recruited in this study. All participants were asked to complete the following questionnaires: Smartphone Addiction Inventory (SPAI), Tri-dimensional personality questionnaire (TPQ), and Chinese Pittsburgh Sleep Questionnaire Index (CPSQI). The results showed that people with a high tendency toward novelty seeking (NS) as a personality trait, compared to those with lower tendency toward NS, are more likely to become addicted to smartphone use. Moreover, those with a stronger trait of being NS and specific impulsivity factor were found to have higher total scores in the SPAI (*p* < 0.05). In addition, linear regression analysis showed that the individuals with higher scores for withdrawal symptoms on the SPAI and anticipatory worry factor on the TPQ tended to have higher CPSQI total scores (*p* < 0.05). This information may be useful for prevention in individuals with personality traits making them vulnerable to smartphone addiction and for designing intervention programs to reduce intensive smartphone use and programs to increase capability in managing smartphone use.

## 1. Introduction

Interaction with digital technology has become a part of life and has increased gradually around the world [1]. Digital technology plays an important role in allowing people to connect with their family and make friends, thus receiving family and social support [2]. Smartphones are typical touch-screen devices with multiple applications (apps) offering quick access to the internet and facilitating message transmissions and communication, but extensive use of smartphones could cause negative psychological effects [3,4,5,6].

The history of smartphones can be traced back to 1992, and they have changed much since. With this evolution, and accompanied by the internet, the mobile phone has become a more capable device, and smart devices with multiple functions have been named smartphones since 2007 [7]. Due to the multiple functionality of a smartphone, the smartphone has become a necessity for individuals, but maladaptive and problematic patterns of smartphone use on school campuses have increased recently [8,9].

Adverse consequences caused by overuse or dependence on smartphones can be easily seen nowadays. For example, those walking across streets and watching videos on a smartphone without checking traffic signals may be in danger of being hit by a car, while driving a car while fumbling with a smartphone may cause a car accident. In addition, students find it more difficult to concentrate in class, resulting in decreased academic performance and productivity while using a smartphone [10,11]. As a smartphone is a powerful portable computer with internet access, able to provide real-time information, smartphone dependency at work or school seems to have become common.

Overuse of smartphones or problematic smartphone use has brought our attention to their relation to addiction [12], although the term “smartphone addiction” is not recognized as a clinical diagnosis in either the DSM-5 [13] or the ICD-11 [14]. The adverse consequences of problematic smartphone use have increased, and problematic behavioral patterns in relation to addiction have been noted.

The central definition of addiction is that individuals depend on a substance or activity [15], although the concept of addiction is difficult to define. Some researchers have stated that behavioral addiction, such as internet addiction, is similar to drug addiction with different forms. Drug addicts most often have physical signs, but these are absent in behavioral addicts, who mostly exhibit behavioral changes and feelings brought by the relevant action [16].

Many aspects of problematic smartphone use seem to share similarities with behavioral addictions; gambling disorder is the only recognized behavioral addiction in DSM-5, while the other behavioral addictions, such as internet gaming and exercise addiction, are classified as impulsive disorders [13]. In addition, several researchers have reported the similar symptoms and characteristics of addiction applicable to problematic smartphone use: people addicted to smartphones have been reported to have characteristics of reduced interest in face-to-face relationships, loss of self-control, preoccupation with smartphone use, withdrawal symptoms, and negative effects on their psychological and social lives [10,17,18]. Such consequences caused by smartphone addiction, where people show recurrent failure to control their behavior of smartphone use with functional impairment or psychological distress, meet the criteria of behavioral addiction proposed by Kardefelt [19].

### 1.1. Personality and Smartphone Addiction

Personality traits such as anxiety, conscientiousness, openness, and emotional stability have been reported as important factors of problematic smartphone use [20,21]. Another commonly studied personality trait, neuroticism, has also been reported to be positively associated with internet addiction [22,23]. A similar finding was reported for predicting smartphone addiction: neuroticism is a positive predictor while agreeableness and conscientiousness are negative predictors among undergraduate students [24]. Besides previous studies on neuroticism and its robust relation with internet addiction and smartphone use disorders, social anxiety and impulsivity were also reported to be correlated to smartphone addiction [25,26]. Briefly, so far, individuals with neuroticism and those who are more anxious about internet activity are more vulnerable to internet addiction [27].

### 1.2. Smartphone Addiction and Sleep Quality

Although depending on smartphones may enhance people’s performance in some ways, such as by browsing information, making responses as a team, and having quick discussions about a project, when it turns to addiction, the perceived performance tends to diminish. Moreover, because of the necessity and multiple functions of a smartphone, people seem to do everything with it, including working and social interaction, and they become more anxious when they are without a smartphone [28]. Due to smartphone dependence, using a smartphone all day can influence people’s sleep, since they have difficulty putting it down before going to bed and have poor sleep quality [29].

A large population survey in the UK examining the correlation between smartphone addiction and poor sleep quality found that approximately 61.6% of participants reported poor sleep, and in the people with smartphone addiction, 68.7% had poor sleep quality [29]. They found that among the people with smartphone addiction, approximately 70% had poor sleep quality, while among those without smartphone addiction, less than 69% reported poor sleep. In addition, problematic internet use has been reported to have an impact on sleep quality because of reduced rapid eye movement (REM) sleep, slow-wave sleep, and sleep efficiency [30]. Moreover, a possible mechanism could be that melatonin secretion is suppressed due to the light of a screen, delaying the beginning of sleep [31].

### 1.3. Smartphone Addiction and Gender

As smartphones have become a necessity, some researchers have reported gender differences in smartphone addiction in different student populations, although these differences are inconsistent [29,32,33]. Increasing smartphone usage was noticed in a parent–children relationship study among elementary school students in Korea, where it was found that 80.4% of participants started smartphone use below 10 years old. They found that 79.2% of Korean children have smartphones, and among their participants, approximately 14.3% were at risk of becoming smartphone addicts; boys had a higher risk of being smartphone addicts than girls, but the difference was not statistically significant [34]. In addition, with age, the prevalence of smartphone addiction increased, because of more and more adolescent owners [35]. Moreover, females spent more time using smartphones than did the males [35], but some researchers noticed the opposite phenomenon [32,34]. Moreover, male and female adolescents were reported to have different behavioral patterns using smartphones: females were more likely to use social media applications on smartphones, while males spent more time gaming and shopping on smartphones [35,36]. Similar smartphone behavioral patterns were found in medical college students, where females were more likely to use multiple media applications while males used game apps [32]. Moreover, gender was reported as moderating smartphone addiction and the perfectionism trait: males with a higher tendency of perfectionism have a higher risk of addiction to smartphone use [37], and the procrastination trait was correlated with smartphone addiction in both gender groups [38].

In brief summary, smartphone addiction causes poor sleep quality, and neuroticism seems the most-studied related personality trait correlated with smartphone addiction. In addition, there seem to be gender differences in smartphone addiction, so the gender effect should be considered. This study proposed to examine the possible factors of smartphone addiction related to personality traits from a neurochemical view and sleep quality, with the gender effect counted as a secondary study aim. Previous studies have reported a similarity between drug and behavioral addiction, and smartphone addiction or so-called internet addiction/technology addiction has been defined as one type of behavioral addiction [39,40]. Both types of addiction indirectly affect the brain system, for example, through the ‘dopaminergic system’, which is involved in the development and maintenance of addiction [39].

The TPQ was developed by Cloningers [41,42] with three dimensions of human temperament: Novelty Seeking (NS), Harm Avoidance (HA), and Reward Dependence (RD). Here, NS is related to intense excitement in relation to novel stimuli, HA is the tendency to respond to aversive stimuli, and RD is the tendency to respond to reward signals. The proposal of NS is related to the dopaminergic system, while HA is related to serotonin [41]. This personality model has been widely used for analysis of addictive behavior [43,44].

A study comparing adolescents with drug addiction and internet addiction (IAD) in relation to personality using the Tridimensional Personality Questionnaire (TPQ) found that the behavioral addicts (IAD) had a higher harm avoidance score than did the groups with substance use disorders (SUD) [45]. Individuals with internet addiction have been found to have high tendency to NS and HA; another study revealed similar personality characteristics among Indonesian smartphone addicts (ISPA) [46]. Each dimension of the TPQ contains four factors, and the specific factor related to smartphone addiction is so far unclear.

In addition, dopamine has been suggested for its involvement in sleep and waking modulations [47], while serotonin is also involved in rapid eye movement (REM) [48]. Using the TPQ, a neurobiochemical-based personality questionnaire was proposed to study smartphone addiction and sleep quality in the current study.

## 2. Materials and Methods

This study was approved by the Human Research Ethics Committee (REC) of the University. All participants signed an informed consent form before participating in the study.

### 2.1. Participants

Participants studying at university or college were recruited and invited to complete the questionnaires with their agreement in entering this study. In total, 422 participants (mean age 20.22 ± 2.34 years, 111 male and 364 female participants) self-reporting no personal history of neurological or psychiatric disorders or cardiovascular diseases were included and required to complete all the questionnaires.

### 2.2. Measurements

Each participant was required to complete the Beck Depression Inventory (BDI) [49,50,51] and Beck Anxiety Inventory (BAI) [52] to screen out the possibility of having mood disorders, such as depressive disorder or anxiety disorder. Due to the comorbidity between depression, anxiety, and smartphone addiction [53], although the causal relationship is unclear, the possibility of having depression or anxiety disorder was excluded using the BDI and BAI as screening instruments. The Chinese version of the BDI was validated and with good split-half reliability of 0.91, so it is suited to screening for the diagnosis of major depressive disorder [51]. In addition, the Chinese version of Beck Anxiety Inventory was validated with the Hamilton Anxiety Rating Scale (HAM-A) [54] with good split-half reliability of 0.91. It is thus promising as a scale for discriminating between anxious and non-anxious people [52]. The BDI and BAI have high internal consistency with Cronbach’s α values of 0.94 and 0.95, respectively. The test–retest reliability values after one week for the BDI and BAI have been reported as r = 0.93 and r = 0.75, respectively [51,55]. In addition, the participants were required to fill in the State-Trait Anxiety Inventory (STAI) [56,57,58], because the anxiety-state and anxiety-trait have been suggested to have an association with psychological well-being and mental illness [57]. In addition, using the STAI, it has been suggested that the state and trait of anxiety mediate alexithymia among Chinese university students [58,59]. Moreover, university students may have demanding lives and experience stress that may affect their use of the internet and social media, such as Facebook [60]. The trait of anxiety was found as a possible predictor for FB use, and an association between gender and anxiety state was reported [60].

#### 2.2.1. Chen’s Smartphone Addiction Inventory

The Smartphone Addiction Inventory (SPAI) [61,62] was used to screen and evaluate the possible diagnostic criteria of smartphone addiction. The SPAI was developed as a self-administered scale with 26 items, divided among four factors: compulsive behavior (SPAI-com), functional impairment (SPAI-FI), withdrawal (SPAI-with.), and tolerance (SPAI-tol.). The test–retest reliability of the SPAI ranges from 0.74 to 0.91 with internal consistency of Cronbach’s α = 0.94.

#### 2.2.2. Tri-Dimensional Personality Questionnaire (TPQ)

The Tri-dimensional Personality Questionnaire (TPQ) was established by Clonninger (1978) [42]. Three dimensions of personality and temperament—Novelty Seeking (NS), Harm Avoidance (HA), and Reward Dependence (RD)—were suggested based on the biological model. The RD dimension has relatively low reliability compared to the other two dimensions, so the RD dimension was not included in the current study. The NS dimension represents the four factors NS1 (exploratory excitability), NS2 (impulsivity), NS3 (extravagance), and NS4 (disorderliness), while the HA dimension contains the four factors HA1 (anticipatory worry), HA2 (fear of uncertainty), HA3 (shyness with strangers), and HA4 (fatigability and asthenia); these have good reliability and were used in the current study [63].

#### 2.2.3. Chinese Version of the Pittsburgh Sleep Quality Index (CPSQI)

The Chinese version of the Pittsburgh Sleep Quality Index (CPSQI) contains 7 subscales: subjective sleep quality, sleep latency, sleep duration, sleep efficiency, sleep disturbance, use of sleep medicine, and daytime dysfunction. It has overall great reliability for all subscales of 0.82–0.83 and acceptable test–retest reliability over a 14–21-day interval with a coefficient of 0.85 for all subjects [64]. The CPSQI has been used to test the sleep quality in a university student population, even though the use of sleep medication may not be suitable for this population [65,66,67].

#### 2.2.4. Statistical Analysis

Chi-square analysis was conducted to compare the nominal variables, such as gender, between groups. In addition, Pearson’s r correlation analysis was conducted to explore the associations among variables. One-way analysis of variance (ANOVA) was conducted to compare the variable differences between groups. Moreover, logistic regression analysis was carried out to explore the potential personality traits associated with smartphone addiction. SPSS 22.0 (SPSS Inc., Chicago, IL, USA) was used for statistical analysis.

## 3. Results

In total, 422 participants with mean age 20.22 (SD = 2.34 years), all university students aged above 18 years, were recruited via online advertisement and posters. Of all the participants, 79 were males with mean age 20.92 ± 1.39 years, and the remaining 343 were females with mean age 20.89 ± 1.37 years. Most of the participants were in the range of minimal to borderline depression or anxiety (BDI = 9.14 ± 7.93, BAI = 7.62 ± 6.31). In addition, no significant difference was found regarding anxiety states or traits between groups (*ps* > 0.05).

### 3.1. Prevalence of Smartphone Addiction

Those participants whose SPAI scores were above the screening cut-off were defined as smartphone addicts (SPA). The cut-off score of 25 in the short form of the SPAI [61,62] was used to identify those who may fit the diagnostic criteria of smartphone addiction. The overall proportion of SPA was 38.63%, including 20.9% of males and 79.1% of females, who showed a high tendency of addiction to smartphones. No significant difference regarding gender was found between the SPA and non-SPA groups (χ^2^ = 0.80, *p* = 0.37).

#### 3.1.1. Comparisons between Smartphone Addiction and Non-Smartphone Addiction

In order to investigate whether the groups differed, a multivariate analysis of variance (MANOVA) was conducted with the screened group as the independent variable and the TPQ dimensions (NS and HA) and CPSQI total score as the dependent variables. The results showed a main effect of group (F = 9.25, *p* < 0.0005), wherein the SPA had significantly poorer sleep quality and higher scores on NS and HA, both dimensions of the TPQ, than did the non-SPA. In further study regarding the factors of NS, HA, and subscales of the PSQI, the results showed a significant main effect of the screened group (F = 3.04, *p* < 0.0005). In addition, univariates showed significance for C1, C5, and C7 of the CPSQI; NS2 (impulsivity) and NS4 (disorderliness) of NS; and HA2 (fear of uncertainty) and HA3 (shyness with strangers) of the HA dimension of the TPQ (*ps* < 0.05) (Table 1).

Pearson’s r correlation analysis was conducted to study the relationships among variables. Significant correlations were found between SPAI subscores and NS and HA, as well as CPSQI total score. In addition, among the specific CPSQI indices, subjective sleep quality (C1), sleep disturbance (C5), and daytime function (C7) were found as possible consequences of smartphone addiction (Table 2).

Further, logistic regression analysis showed that the subscales of disorderliness under NS and fear of uncertainty under HA were positive predictors for SPA. Anticipatory worry under HA and withdrawal symptoms could be predictors for the total sleep quality score (Table 3).

#### 3.1.2. Study of Gender Effects on the Associations among Smartphone Addiction, Personality, and Sleep Quality

Due to previous reports of different prevalence rates of smartphone addiction between genders, we further studied the differences between SPA and non-SPA across genders, and we split the comparisons between genders. The results showed a significant main effect of the screened group (F_m_ = 3.96, *p* = 0.01; F_F_ = 7.02, *p* < 0.0005) in which the SPA group significantly had poorer sleep quality and higher scores on NS and HA in both gender groups. Further univariates showed significance for only HA2 (fear of uncertainty) in the male SPA group; the significantly higher scores of C1, C5, and C7 of the CPSQI, NS2 and NS4 under NS, and HA2 and HA3 under HA remained significant in the female SPA group (*p* < 0.05) (Table 4), but not in the male group (*ps* > 0.05).

Moreover, among the SPAI subscales, TPQ dimensions, and indices of sleep quality across gender, we found that the correlations between three SPAI components (compulsivity, withdrawal, and functional impairment) and NS2 (impulsivity) remained significant in both gender groups, except that the association between SPAI-FI and NS2 was remained significantly only in the female group. The correlation between SPAI component scores and NS4 remained in the female SPA group, but not in the male SPA group. In addition, the correlations between HA3 and HA4 and PSAI component scores remained significant in the female group only.

## 4. Discussion

The findings of the current study demonstrate the facts relating to a higher proportion of smartphone addiction (or problematic smartphone use) [29]. In addition, with addiction to smartphones, higher risk of psychological distress and poor sleep quality was found, which is inconsistent with a previous report that more and more young adults report poor sleep quality in a higher percentage when they become addicted to smartphones [29].

In addition, higher NS and HA scores were found in the SPA group compared to the non-SPA group, which is in agreement with previous studies [45,46] reporting that addicts tend to have higher NS and HA scores than non-addicts. Moreover, the impulsivity and disorderliness factors of NS were found to be related to the SPA group, which demonstrated that a high risk of being SPA may be caused by poor impulse control [68,69,70]. The other subscale of NS, disorderliness, was also found to be higher in the SPA than in the non-SPA group, which may represent a phenomenon of smartphone dependence wherein the SPA group have difficulty in organizing their life and easily become addicted [71]. This association was similar to the finding for abstinent substance abuse [72], implying difficulties for individuals who are vulnerable to addiction.

Moreover, higher scores on the subscales of fear of uncertainty and shyness under HA were found in the SPA group, indicating that the SPA may use the device applications, such as social media, and the smartphone as a facility for them to develop social interaction. This finding supports a previous report that people who tend to develop interpersonal relations online and are anxious about events online are more likely to become addicted [27].

Furthermore, the positive correlations between four components (compulsivity, withdrawal, tolerance, and functional impairment) of the SPAI and the sum scores of NS and HA, especially impulsivity and disorderliness under NS, along with fear of uncertainty and shyness with strangers, both subscales of HA, imply a possible psychopathology of smartphone addiction [73].

Furthermore, poor sleep quality was reported more in the SPA group than in the non-SPA group, which is consistent with previous studies [29], although the difference was not significant. A possible explanation could be that the participants we recruited were in their term examination period; during such a period, students have a higher tendency to stay up late for their exam preparation. Moreover, the significant associated factors we found as positive predictors of their sleep quality were the withdrawal component of SPAI and the subscale of anticipator worry, which represent similar behavioral impacts of withdrawal symptoms on the quality of their sleep [74].

The rapidly increased use of smartphones may result in a high prevalence of smartphone addiction and sleep disturbance among university students [29]. This would cause not only physical, but also psychological health problems. Moreover, a correlation was found in female university students where personalities with higher impulsivity (NS2), fear of uncertainty (HA3), and shyness with strangers (HA3) positively correlated with smartphone addiction; this was noticed in the females only. This correlation is consistent with a previous report that females have higher impulsivity with poor self-control and are more likely to become smartphone addicts [73].

There were a few limitations in the current study. One limitation was that no specific devices of smartphone use were categorized to investigate the correlation between personality dimensions and smartphone use and the causes of poor sleep quality in SPA. In addition, our data were limited by self-report measurements, so the validity may be contingent on the accuracy of their reports. Furthermore, this would be specific cultural population and the results may not be generalizable to all population. The numbers of participants in the different gender groups were unequal, which may limit the generalization of results across genders.

Future research may continue to look at the mechanism between personality traits and smartphone use and its impact on sleep quality. In addition, sleep quality was recorded by self-report, which may have limited generalizability; a further study measuring sleep quality using a laboratory design or sleep polysomnography examination may provide more objective data to investigate more specific components of sleep impacted by excessive smartphone use.

## 5. Conclusions

Being too dependent on smartphones, or addicted to smartphones, has consequent adverse effects on sleep quality, specific sleep latency, and daytime dysfunction. In addition, those with personality traits of tendency toward novelty seeking and harm avoidance are more vulnerable to becoming smartphone addicts. Moreover, people who are more disorderly and fear strangers have a higher risk of coming to rely on smartphone use. Furthermore, more withdrawal symptoms of smartphone addiction and the anticipatory worry personality trait could predict poor sleep quality in individuals.

The reported findings show a possible neurochemical mechanism from personality traits to smartphone addiction and gender differences; this contributes to the frameworks and theoretical developments in smartphone addiction, providing implications for prevention and intervention to reduce smartphone addiction among university students.

## Figures and Tables

**Table 1:** Comparisons between the SPA and non-SPA groups.

	Non-SPA (*n* = 163)		SPA (*n* = 259)		Statistics (*p)*
	Mean	S.D	Mean	S.D	
Age	21.03	1.35	20.81	1.41	
Gender (Male/Female)	34/129		46/213		0.80 (0.37)
BDI	10.50	8.30	8.31	7.68	1.13 (0.29
BAI	8.50	6.15	7.08	6.42	0.75 (0.39)
STAI_state	41.42	5.95	40.04	10.73	1.93 (0.17)
STAI_trait	37.93	19.43	37.90	14.77	--
TPQ					
*Novelty Seeking_sum*	16.80	4.03	15.78	3.93	6.90 (0.009)
*Exploratory excitability*	2.84	1.23	2.71	1.28	1.13 (0.29)
*Impulsivity*	6.27	2.02	0.74	1.89	7.53 (0.006)
*Extravagance*	3.89	1.60	3.85	1.63	0.07 (0.79)
*Disorderliness*	1.56	1.18	1.22	1.12	9.12 (0.003)
*Harm Avoidance_sum*	14.3	1.92	12.92	4.47	10.72 (0.001)
*Anticipatory worry*	4.67	1.92	4.42	1.74	2.02 (0.16)
*Fear of uncertainty*	2.73	1.41	2.12	1.38	19.08 (<0.0005)
*Shyness*	2.67	1.26	2.33	1.36	6.46 (0.01)
*Fatigability and asthenia*	4.26	1.74	4.05	1.85	1.45 (0.23)
CPSQI					
*C1—subjective sleep quality*	1.26	0.73	1.09	0.70	6.11 (0.01)
*C2—sleep latency*	1.75	0.97	1.59	0.89	3.31 (0.07)
*C3—sleep duration*	2.61	0.98	2.58	1.01	0.08 (0.78)
*C4—sleep efficiency*	2.71	0.85	2.64	0.92	1.59 (0.44)
*C5—sleep disturbance*	1.22	0.71	1.08	0.67	3.92 (0.048)
*C6—use of sleep medicine*	0.25	0.59	0.25	0.60	--
*C7—daytime dysfunction*	1.15	0.77	0.90	0.69	12.49 (<0.0005)

Note: SPA: smartphone addicts, Non-SPA: non-smartphone addicts; BDI: Beck Depression Inventory; BAI: Beck Anxiety Inventory; STAI: State–Trait Anxiety Inventory; TPQ: Tridimensional Personality Questionnaire; CPSQI: Chinese Pittsburgh Sleep Quality Index.

**Table 2:** The correlational matrix relating TPQ subscales, SPAI, and sleep quality.

	Mean	S.D	1.	2.	3.	4.	5.	6.	7.	8.	9.	10.	11.	12.	13.	14.	15.	16.	17.	18.	19.	20.	21.	22.	23.	24.	25.
1.	60.21	13.54	--																								
2.	20.44	4.96	0.94 ***	--																							
3.	14.91	3.51	0.90 ***	0.82 ***	--																						
4.	7.20	1.87	0.78 ***	0.66 ***	0.65 ***	--																					
5.	17.66	4.59	0.92 ***	0.81 ***	0.74 ***	0.68 ***	--																				
6.	9.14	7.93	0.22	0.27 *	0.07	0.07	0.24	--																			
7.	7.62	6.31	0.24	0.26 *	0.16	−0.02	0.74 ***	0.74 ***	--																		
8.	16.18	4.00	0.17 **	0.16 *	0.14 *	0.15 *	0.16 **	−0.11	−0.20	--																	
9.	2.76	1.26	0.08	0.07	0.08	0.05	0.07	−0.02	−0.16	0.56 ***	--																
10.	5.94	1.96	0.19 ***	0.20 ***	0.20 ***	0.09	0.16 **	−0.20	−0.22	0.73 ***	0.28 ***	--															
11.	3.86	1.62	0.06	0.04	0.03	0.09	0.07	−0.17	−0.18	0.63 ***	0.08	0.27 ***	--														
12.	1.35	1.15	0.11 *	10.0 *	0.00	0.14 *	0.14 *	0.16	0.11	0.48 ***	0.13 *	0.14 ***	0.21 ***	--													
13.	13.47	4.38	0.15 *	0.14 *	0.13 *	0.14 *	0.12 *	−0.43 ***	−0.34 *	0.36 ***	0.22 ***	0.33 ***	0.17 ***	0.18 ***	--												
14.	4.52	1.82	0.20	0.02	0.10	0.07	0.00	−0.45 ***	−0.37 *	0.29 ***	0.18 ***	0.17 **	0.21 ***	0.12 *	0.72 ***	--											
15.	2.36	1.42	0.16 **	0.16 *	0.16 **	0.11 *	0.13 *	−0.10	0.01	0.29 ***	0.16 *	0.30 ***	0.12 *	0.19 ***	0.61 ***	0.20 ***	--										
16.	2.46	1.33	0.12 *	0.12 *	0.15 *	0.13 *	0.15 *	−0.32 *	−0.22	0.19 ***	0.14 *	0.25 ***	0.02	0.04	0.64 ***	0.26 ***	0.31 ***	--									
17.	4.13	1.81	0.11 *	0.11 *	0.08	0.09	0.08	−0.43 ***	−0.36 *	0.21 ***	0.13 *	0.20 ***	0.10 *	0.14 *	0.76 ***	0.39 ***	0.27 ***	0.32 ***	--								
18.	9.32	2.02	0.21 ***	0.21 ***	0.22 ***	0.10 *	0.21 ***	0.41 **	0.31 *	−0.03	0.00	0.07	−0.08	−0.10 *	−0.04	−0.16 *	0.08	0.06	−0.05	--							
19.	1.16	0.71	0.14 *	0.14 *	0.11 *	0.10 *	0.14 *	0.54 ***	0.36 *	−0.02	−0.15 *	−0.00	0.02	0.09	−0.04	−0.06	0.02	−0.01	−0.04	0.49 ***	--						
20.	1.65	0.92	1.0 *	10.0 *	0.08	0.16 **	0.11 *	0.26 *	0.25 *	0.03	−0.09	−0.01	0.07	0.16 **	0.06	−0.06	0.12 *	0.01	0.11 *	0.45 ***	0.33 ***	--					
21.	2.59	1.00	0.04	0.04	10.0 *	−0.10 *	−0.00	−0.06	−0.22	−0.03	0.15 *	0.10 *	−0.14 *	−0.31 ***	−0.06	−0.07	−0.02	0.11 *	−0.13 *	0.50 ***	−0.12 *	−0.13 ***	--				
22.	2.66	0.89	0.06	0.06	0.09	−0.07	0.04	0.09	0.07	−0.08	0.15 *	0.05	−0.17 ***	−0.33 ***	−10.0 *	−0.11 *	−0.04	0.06	−0.14 *	0.55 ***	−0.12 *	−0.21 ***	0.85 ***	--			
23.	1.14	0.69	0.16 *	0.15 *	0.19 ***	0.07	0.14 *	−0.11	−0.11	0.06	0.03	0.05	0.05	−0.06	0.02	0.01	0.03	0.08	−0.04	0.48 ***	0.21 ***	−0.02	0.48 ***	0.36 ***	--		
24.	0.25	0.60	−0.06	−0.08	−0.13 *	0.05	−0.03	−0.18	−0.11	0.05	−0.12 *	−0.08	0.18 ***	0.30 ***	0.08	0.09	0.03	−0.06	0.13 *	−0.30 ***	0.08	0.24 ***	−0.74 ***	−0.73 ***	−0.30 ***	--	
25.	1.00	0.73	0.24 ***	0.23 ***	0.23 ***	0.15 *	0.24 ***	0.28 *	0.32 *	0.00	−0.01	0.08	−0.07	0.02	−0.02	−0.13 *	0.14 *	−0.01	−0.01	0.59 ***	0.22 ***	0.14 *	0.15 *	0.12 *	0.31 ***	−0.11 *	--

Note: 1. Total score of Smartphone Addiction Inventory (SPAI), 2. Subscale, compulsivity of SPAI; 3. Subscale, Withdrawal of SPAI; 4. Subscale, tolerance of SPAI; 5. Subscale, Functional Impairment of SPAI; 6. Beck Depression Inventory; 7. Beck Anxiety Inventory; 8. Total score of Novelty Seeking subscale of Tridimensional Personality Questionnaire (TPQ); 9. exploratory excitability subscale of NS; 10. impulsivity subscale of NS; 11. extravagance subscale of NS; 12. disorderliness subscale of NS; 13. total score of Harm Avoidance (HA) of TPQ; 14. Subscale, anticipatory worry of HA; 15. Subscale, fear of uncertainty of HA; 16. Subscale, shyness with strangers of HA; 17. Subscale, fatigability & asthenia of HA; 18. total score of Chinese version of Pittsburgh Sleep Quality Index (CPSQI); 19. C1 component-subjective sleep quality of CPSQI; 20. C2 component-sleep latency of CPSQI; 21. C3 component-sleep duration of CPSQI; 22. C4 component-sleep efficiency of CPSQI; 23. C5 component-sleep disturbance of CPSQI; 24. C6 component-using sleep medicine of CPSQI; 25. C7 component-daytime function of CPSQI; * *p*-value < 0.05; ** *p*-value < 0.001; *** *p*-value < 0.0005.

**Table 3:** Multiple regression analysis model coefficients for sleep quality with a smartphone addiction component and personality subscales.

Variables/CPSQI_Total	Model 1			F (*p*)	Model 2			F (*p*)	Model 3			F (*p*)
	B	SE_B_	β		B	SE_B_	β		B	SE_B_	β	
SPAI_with.	0.13	0.03	0.22	21.62 (<0.0005)	0.13	0.03	0.22	16.85 (<0.0005)	0.12	0.03	0.05	13.01 (<0.0005)
HA_anti.					−0.18	0.05	−0.16		−0.19	0.05	−0.17	
age									0.16	0.05	0.07	

Note: CPSQI_total: total score of Chinese version of Pittsburgh Sleep Quality Index; SPAI_with.: the subscale, withdrawal of Smartphone Addiction Inventory (SPAI); HA_anti: the subscale, anticipatory worry of Harm Avoidance factor.

**Table 4:** Comparisons between SPA and non-SPA in the female group.

	Non-SPA (*n* = 129)		SPA (*n* = 214)		Statistics (*p)*
	** Mean **	**S.D**	** Mean **	**S.D**	
Age	20.81	1.40	21.02	1.38	1.92 (0.17)
BDI	8.44	8.00	10.90	8.78	1.14 (0.29)
BAI	7.24	6.71	8.95	6.45	0.88 (0.35)
STAI_state	39.26	9.54	40.59	5.48	1.77 (0.18)
STAI_trait	42.44	6.91	42.45	16.06	0.003 (0.96)
TPQ					
*Novelty Seeking_sum*	15.61	3.93	16.78	3.97	7.05 (0.008)
*Exploratory excitability*	2.63	1.26	2.79	1.14	1.48 (0.23)
*Impulsivity*	5.69	1.87	6.30	2.03	8.07 (0.005)
*Extravagance*	3.86	1.64	3.92	1.66	0.10 (0.75)
*Disorderliness*	1.19	1.09	1.5	1.17	8.31 (0.004)
*Harm Avoidance_sum*	12.87	4.45	13.93	3.98	4.91 (0.03)
*Anticipatory worry*	4.34	1.72	4.47	1.88	0.47 (0.49)
*Fear of uncertainty*	2.16	1.37	2.67	1.42	11.14 (0.001)
*Shyness*	2.33	1.31	2.60	1.24	3.55 (0.06)
*Fatigability and asthenia*	4.05	1.85	4.19	1.76	0.44 (0.51)
CPSQI					
*C1—subjective sleep quality*	1.10	0.70	1.27	0.72	4.61 (0.03)
*C2—sleep latency*	1.63	0.89	1.79	0.93	2.49 (0.12)
*C3—sleep duration*	2.54	1.06	2.57	1.01	2.49 (0.12)
*C4—sleep efficiency*	2.61	0.96	2.65	0.91	0.14 (0.71)
*C5—sleep disturbance*	1.11	0.68	1.22	0.70	2.15 (0.14)
*C6—use of sleep medicine*	0.25	0.61	0.28	0.64	0.15 (0.70)
*C7—daytime dysfunction*	0.93	0.68	1.20	0.76	12.07 (0.001)

## References

[1] Giedd J.N. (2012). The digital revolution and adolescent brain evolution. J. Adolesc. Health.

[2] Gemmill E.L., Peterson M. (2006). Technology Use Among College Students: Implications for Student Affairs Professionals. NASPA J..

[3] Alavi S.S., Maracy M.R., Jannatifard F., Eslami M. (2011). The effect of psychiatric symptoms on the internet addiction disorder in Isfahan’s University students. J. Res. Med. Sci..

[4] Folaranmi A. (2013). A Survey of Facebook Addiction Level among Selected Nigerian University Undergraduates. New Media Mass Commun..

[5] Kim K., Ryu E., Chon M.-Y., Yeun E.-J., Choi S.-Y., Seo J.-S., Nam B.-W. (2006). Internet addiction in Korean adolescents and its relation to depression and suicidal ideation: A questionnaire survey. Int. J. Nurs. Stud..

[6] Shapira N.A., Goldsmith T.D., Keck P.E., Khosla U.M., McElroy S.L. (2000). Psychiatric features of individuals with problematic internet use. J. Affect. Disord..

[7] Andrew O. (2018). The History and Evolution of the Smartphone: 1992–2018. https://www.textrequest.com/blog/history-evolution-smartphone/.

[8] Amidtaher M., Saadatmand S., Moghadam Z., Fathi G., Afshar R. (2016). The Relationship between Mobile Cellphone Dependency, Mental Health and Academic Achievement. Am. J. Educ. Res..

[9] Campbell S.W., Park Y.J. (2008). Social Implications of Mobile Telephony: The Rise of Personal Communication Society. Sociol. Compass.

[10] Duke E., Montag C. (2017). Smartphone addiction, daily interruptions and self-reported productivity. Addict. Behav. Rep..

[11] Grant J.E., Lust K., Chamberlain S.R. (2019). Problematic smartphone use associated with greater alcohol consumption, mental health issues, poorer academic performance, and impulsivity. J. Behav. Addict..

[12] De-Sola Gutiérrez J., Rodríguez de Fonseca F., Rubio G. (2016). Cell-Phone Addiction: A Review. Front. Psychiatry.

[13] American Psychiatric Association (2013). Diagnostic and Statistical Manual of Mental Disorders.

[14] WHO (2018). International Statistical Classification of Diseases and Related Health Problems.

[15] Widyanto L., McMurran M. (2004). The psychometric properties of the internet addiction test. Cyberpsychol. Behav..

[16] Alavi S.S., Ferdosi M., Jannatifard F., Eslami M., Alaghemandan H., Setare M. (2012). Behavioral Addiction versus Substance Addiction: Correspondence of Psychiatric and Psychological Views. Int. J. Prev. Med..

[17] Lanaj K., Johnson R.E., Barnes C.M. (2014). Beginning the workday yet already depleted? Consequences of late-night smartphone use and sleep. Organ. Behav. Hum. Decis. Process..

[18] Lin Y.-H., Lin Y.-C., Lee Y.-H., Lin P.-H., Lin S.-H., Chang L.-R., Tseng H.-W., Yen L.-Y., Yang C.C.H., Kuo T.B.J. (2015). Time distortion associated with smartphone addiction: Identifying smartphone addiction via a mobile application (App). J. Psychiatr. Res..

[19] Kardefelt-Winther D., Heeren A., Schimmenti A., van Rooij A., Maurage P., Carras M., Edman J., Blaszczynski A., Khazaal Y., Billieux J. (2017). How can we conceptualize behavioural addiction without pathologizing common behaviours?. Addiction.

[20] Hussain Z., Griffiths M.D., Sheffield D. (2017). An investigation into problematic smartphone use: The role of narcissism, anxiety, and personality factors. J. Behav. Addict..

[21] Monacis L., Griffiths M.D., Limone P., Sinatra M., Servidio R. (2020). Selfitis Behavior: Assessing the Italian Version of the Selfitis Behavior Scale and Its Mediating Role in the Relationship of Dark Traits with Social Media Addiction. Int. J. Environ. Res. Public Health.

[22] Andreassen C.S., Griffiths M.D., Gjertsen S.R., Krossbakken E., Kvam S., Pallesen S. (2013). The relationships between behavioral addictions and the five-factor model of personality. J. Behav. Addict..

[23] Tsai H.F., Cheng S.H., Yeh T.L., Shih C.C., Chen K.C., Yang Y.C., Yang Y.K. (2009). The risk factors of Internet addiction—A survey of university freshmen. Psychiatry Res..

[24] Erdem C., Uzun A.M. (2020). Smartphone Addiction Among Undergraduates: Roles of Personality Traits and Demographic Factors. Technol. Knowl. Learn..

[25] Peterka-Bonetta J., Sindermann C., Elhai J.D., Montag C. (2019). Personality Associations with Smartphone and Internet Use Disorder: A Comparison Study Including Links to Impulsivity and Social Anxiety. Front. Public Health.

[26] Servidio R., Sinatra M., Griffiths M.D., Monacis L. (2021). Social comparison orientation and fear of missing out as mediators between self-concept clarity and problematic smartphone use. Addict. Behav..

[27] Chang Y.H., Lee Y.T., Hsieh S. (2019). Internet Interpersonal Connection Mediates the Association between Personality and Internet Addiction. Int. J. Environ. Res. Public Health.

[28] Li L., Lin T.T.C. (2019). Smartphones at Work: A Qualitative Exploration of Psychological Antecedents and Impacts of Work-Related Smartphone Dependency. Int. J. Qual. Methods.

[29] Sohn S.Y., Krasnoff L., Rees P., Kalk N.J., Carter B. (2021). The Association between Smartphone Addiction and Sleep: A UK Cross-Sectional Study of Young Adults. Front. Psychiatry.

[30] Dworak M., Schierl T., Bruns T., Strüder H.K. (2007). Impact of singular excessive computer game and television exposure on sleep patterns and memory performance of school-aged children. Pediatrics.

[31] Wood A.W., Loughran S.P., Stough C. (2006). Does evening exposure to mobile phone radiation affect subsequent melatonin production?. Int. J. Radiat. Biol..

[32] Chen B., Liu F., Ding S., Ying X., Wang L., Wen Y. (2017). Gender differences in factors associated with smartphone addiction: A cross-sectional study among medical college students. BMC Psychiatry.

[33] Tangmunkongvorakul A., Musumari P.M., Tsubohara Y., Ayood P., Srithanaviboonchai K., Techasrivichien T., Suguimoto S.P., Ono-Kihara M., Kihara M. (2020). Factors associated with smartphone addiction: A comparative study between Japanese and Thai high school students. PLoS ONE.

[34] Lee E.J., Kim H.S. (2018). Gender Differences in Smartphone Addiction Behaviors Associated with Parent–Child Bonding, Parent–Child Communication, and Parental Mediation among Korean Elementary School Students. J. Addict. Nurs..

[35] Taywade A., Khubalkar R. (2019). Gender differences in smartphone usage patterns of adolescents. Int. J. Indian Psychol..

[36] Lee S.Y., Lee D., Nam C.R., Kim D.Y., Park S., Kwon J.G., Kweon Y.S., Lee Y., Kim D.J., Choi J.S. (2018). Distinct patterns of Internet and smartphone-related problems among adolescents by gender: Latent class analysis. J. Behav. Addict..

[37] Yang W., Morita N., Zuo Z., Kawaida K., Ogai Y., Saito T., Hu W. (2021). Maladaptive Perfectionism and Internet Addiction among Chinese College Students: A Moderated Mediation Model of Depression and Gender. Int. J. Environ. Res. Public Health.

[38] Yang X., Wang P., Hu P. (2020). Trait Procrastination and Mobile Phone Addiction among Chinese College Students: A Moderated Mediation Model of Stress and Gender. Front. Psychol..

[39] Holden C. (2001). ‘Behavioral’ addictions: Do they exist?. Science.

[40] Roberts J.A., Yaya L.H.P., Manolis C. (2014). The invisible addiction: Cell-phone activities and addiction among male and female college students. J. Behav. Addict..

[41] Cloninger C.R. (1987). A systematic method for clinical description and classification of personality variants: A proposal. Arch. Gen. Psychiatry.

[42] Cloninger C.R., Przybeck T.R., Svrakic D.M. (1991). The Tridimensional Personality Questionnaire: U.S. normative data. Psychol. Rep..

[43] Ball S.A. (2005). Personality traits, problems, and disorders: Clinical applications to substance use disorders. J. Res. Personal..

[44] Goudriaan A.E., Oosterlaan J., de Beurs E., Van den Brink W. (2004). Pathological gambling: A comprehensive review of biobehavioral findings. Neurosci. Biobehav. Rev..

[45] Ko C.H., Yen J.Y., Chen C.C., Chen S.H., Wu K., Yen C.F. (2006). Tridimensional personality of adolescents with internet addiction and substance use experience. Can. J. Psychiatry.

[46] Hanafi E., Siste K., Wiguna T., Kusumadewi I., Nasrun M.W. (2019). Temperament profile and its association with the vulnerability to smartphone addiction of medical students in Indonesia. PLoS ONE.

[47] Monti J.M., Monti D. (2007). The involvement of dopamine in the modulation of sleep and waking. Sleep Med. Rev..

[48] Ursin R. (2002). Serotonin and sleep. Sleep Med. Rev..

[49] Beck A.T., Steer R.A. (1984). Internal consistencies of the original and revised Beck Depression Inventory. J. Clin. Psychol..

[50] Beck A.T., Steer R.A., Ball R., Ranieri W. (1996). Comparison of Beck Depression Inventories -IA and -II in psychiatric outpatients. J. Personal. Assess..

[51] Lu M.-L., Che H.H.W., Chang S., Shen W.W. (2002). Reliability and Validity of the Chinese Version of the Beck Depression Inventory-II. Taiwan J. Psychiatry.

[52] Che H.-H., Lu M.-L., Chen H.-C., Chang S.-W., Lee Y.-J. (2006). Translated title of the contribution: Validation of the Chinese Version of the Beck Anxiety Inventory. J. Med..

[53] Matar Boumosleh J., Jaalouk D. (2017). Depression, anxiety, and smartphone addiction in university students—A cross sectional study. PLoS ONE.

[54] Hamilton M. (1959). The assessment of anxiety states by rating. Br. J. Med. Psychol..

[55] Beck A.T., Epstein N., Brown G., Steer R.A. (1988). An inventory for measuring clinical anxiety: Psychometric properties. J. Consult. Clin. Psychol..

[56] Hedberg A.G. (1972). Review of State-Trait Anxiety Inventory. Prof. Psychol..

[57] Shek D.T. (1993). The Chinese version of the State-Trait Anxiety Inventory: Its relationship to different measures of psychological well-being. J. Clin. Psychol..

[58] Shek D.T.L. (1988). Reliability and factorial structure of the Chinese version of the State-Trait Anxiety Inventory. J. Psychopathol. Behav. Assess..

[59] Franzoi I.G., Sauta M.D., Granieri A. (2020). State and Trait Anxiety among University Students: A Moderated Mediation Model of Negative Affectivity, Alexithymia, and Housing Conditions. Front. Psychol..

[60] Xie W., Karan K. (2019). Predicting Facebook addiction and state anxiety without Facebook by gender, trait anxiety, Facebook intensity, and different Facebook activities. J. Behav. Addict..

[61] Lin Y.H., Chang L.R., Lee Y.H., Tseng H.W., Kuo T.B., Chen S.H. (2014). Development and validation of the Smartphone Addiction Inventory (SPAI). PLoS ONE.

[62] Lin Y.H., Pan Y.C., Lin S.H., Chen S.H. (2017). Development of short-form and screening cutoff point of the Smartphone Addiction Inventory (SPAI-SF). Int. J. Methods Psychiatr. Res..

[63] Chen W.J., Chen H.-M., Chen C.-C., Chen C.-C., Yu W.-Y., Cheng A.T. (2002). Cloninger’s Tridimensional Personality Questionnaire: Psychometric properties and construct validity in Taiwanese adults. Compr. Psychiatry.

[64] Tsai P.S., Wang S.Y., Wang M.Y., Su C.T., Yang T.T., Huang C.J., Fang S.C. (2005). Psychometric evaluation of the Chinese version of the Pittsburgh Sleep Quality Index (CPSQI) in primary insomnia and control subjects. Qual. Life Res..

[65] Cheng S.H., Shih C.C., Lee I.H., Hou Y.W., Chen K.C., Chen K.T., Yang Y.K., Yang Y.C. (2012). A study on the sleep quality of incoming university students. Psychiatry Res..

[66] Guo S., Sun W., Liu C., Wu S. (2016). Structural Validity of the Pittsburgh Sleep Quality Index in Chinese Undergraduate Students. Front. Psychol..

[67] Li L., Wang Y.Y., Wang S.B., Zhang L., Li L., Xu D.D., Ng C.H., Ungvari G.S., Cui X., Liu Z.M. (2018). Prevalence of sleep disturbances in Chinese university students: A comprehensive meta-analysis. J. Sleep Res..

[68] Lee H.W., Choi J.S., Shin Y.C., Lee J.Y., Jung H.Y., Kwon J.S. (2012). Impulsivity in internet addiction: A comparison with pathological gambling. Cyberpsychol. Behav. Soc. Netw..

[69] Robbins T.W., Gillan C.M., Smith D.G., de Wit S., Ersche K.D. (2012). Neurocognitive endophenotypes of impulsivity and compulsivity: Towards dimensional psychiatry. Trends Cogn. Sci..

[70] Yucens B., Uzer A. (2018). The relationship between internet addiction, social anxiety, impulsivity, self-esteem, and depression in a sample of Turkish undergraduate medical students. Psychiatry Res..

[71] Kuss D.J., Kanjo E., Crook-Rumsey M., Kibowski F., Wang G.Y., Sumich A. (2018). Problematic Mobile Phone Use and Addiction Across Generations: The Roles of Psychopathological Symptoms and Smartphone Use. J. Technol. Behav. Sci..

[72] Gómez-Perreta C., Pérez C., Portolés M., Salom R. (2001). Tridimensional theory of personality: Applications to substance abuse disorders. Actas Esp. Psiquiatr..

[73] Kim Y., Jeong J.-E., Cho H., Jung D.-J., Kwak M., Rho M.J., Yu H., Kim D.-J., Choi I.Y. (2016). Personality Factors Predicting Smartphone Addiction Predisposition: Behavioral Inhibition and Activation Systems, Impulsivity, and Self-Control. PLoS ONE.

[74] Moattari M., Moattari F., Kaka G., Kouchesfahani H.M., Sadraie S.H., Naghdi M. (2017). Smartphone Addiction, Sleep Quality and Mechanism. Int. J. Cogn. Behav..

